# Comparison of Two Measurement Devices for Pulse Wave Velocity in Children: Which Tool Is Useful to Detect Vascular Alterations Caused by Overweight?

**DOI:** 10.3389/fped.2019.00334

**Published:** 2019-08-20

**Authors:** Julia Elmenhorst, Heidi Weberruss, Martina Mayr, Karin Pfister, Renate Oberhoffer

**Affiliations:** ^1^Department of Pediatric Cardiology and Congenital Heart Defects, German Heart Centre, Munich, Germany; ^2^Faculty of Sport and Health Sciences, Institute of Preventive Pediatrics, Technical University of Munich, Munich, Germany; ^3^Vascular and Endovascular Surgery, University of Regensburg, Regensburg, Germany

**Keywords:** arterial stiffness, children, pulse wave velocity, cardiovascular disease, ultrasound

## Abstract

Vascular alterations may lead to manifest cardiovascular disease in future life. There is a tremendous time delay between the onset and obvious clinical appearance of vascular alterations. Pulse wave velocity (PWV) is one subclinical parameter to detect vascular alterations at a very early stage. Different techniques exist to measure PWV non-invasively as a vascular parameter—all with their own technique-inherent advantages, challenges, and pitfalls. The aim of this study was to compare two techniques to measure PWV, to assess their agreement, and interchangeability. In 780 (♀ = 49.4%) healthy children and adolescents (mean age: 11.61 ± 2.11 years), PWV was obtained with two different techniques. Ultrasound-measured local PWV (PWVβ) at the carotid artery was graphically compared by a Bland–Altman plot with aortic PWV (aPWV), measured oscillometrically on the brachial artery. Reproducibility was assessed with the concordance correlation coefficient by Lin (ρc). Furthermore, participants were categorized by BMI as normal weight (N) or overweight/obese (O) to identify differences in PWVβ and aPWV caused by an increased BMI. Mean PWVβ was lower (4.01 ± 0.44 m/s) than mean aPWV (4.67 ± 0.34 m/s). The two methods differ by mean Δ0.66 ± 0.47 m/s (95% CI: 0.62 to 0.69 m/s; *p* < 0.001). Bland–Altman analysis indicated the 95% limits of agreement (−0.26 to 1.57) without any evidence of systemic difference. Lin's ρc represented a weak concordance between PWVβ and aPWV (ρc = 0.122; 95% CI: 0.093–0.150). There was no difference in PWVβ between N and O, whereas aPWV was higher in O: 4.81 ± 0.42 m/s than in N: 4.65 ± 0.32 m/s (*p* < 0.001). The difference, Δ0.16 m/s, 95% CI [−0.25; −0.08], was significant, *t*_(121)_ = −3.76, *p* < 0.001, with a medium-sized effect. PWVβ (ultrasound) and aPWV (oscillometry) show a level of disagreement that includes clinically important discrepancies. A discrimination between normal and altered vascular function was possible with aPWV but not with PWVβ.

## Introduction

Cardiovascular disease (CVD) is the most common cause of death in Germany and other industrialized nations (1). Among classical risk factors like high blood pressure, smoking, physical inactivity, and obesity (2), vascular parameters like pulse wave velocity (PWV) become more and more important. PWV is the speed at which the pressure wave, generated by the pulsatile ejection of the left ventricle, is transmitted through the arterial system (3). An increased PWV is a surrogate marker for increased vascular stiffness, caused by atherosclerotic changes, and degenerative vascular remodeling (4). Furthermore, increased vascular stiffness is associated with higher systolic blood pressure, increased cardiac afterload (5), and decreased myocardial perfusion pressure (6), which compromises cardiac function. Numerous studies showed that the pathogenic process of CVD begins in early childhood and adolescence (7–9). It is therefore important, to investigate the vascular status of children and adolescents for early detection of vascular stiffness as an independent cardiovascular risk factor (10).

The measurement of PWV is a simple and reproducible method to examine vascular stiffness (11). Therefore, PWV, in addition to intima–media thickness (IMT), is recommended by the European Society of Hypertension/Cardiology (ESH/ESC) to quantify vascular stiffness that caused end-organ damage in patients with arterial hypertension (12). To measure PWV non-invasively, different techniques exist (tonometric, oscillometric, ultrasound-based, with MRI, or via piezo-electronic pressure transducers), all with their own technique-inherent advantages, challenges, and pitfalls (13). Therefore, the aim of this study was to compare ultrasound measured local PWV (PWVβ) at the carotid artery with aortic PWV (aPWV), measured oscillometrically at the brachial artery. Both methods were compared for agreement and reproducibility. The qualities of both methods are of special interest in childhood risk screening: both methods are non-invasive and can be performed within a short time (10–15 min). Furthermore, the oscillometric method is not operator-dependent and as easy to perform as a regular blood pressure measurement. The ultrasound technique can be performed with standard ultrasound equipment plus an additional software package. It could be easily used as a standard procedure in pediatric centers.

## Patients and Methods

### Study Population

PWV measurement was part of the research project “Sternstunden der Gesundheit” in Berchtesgaden, Bavaria, Germany, from October 2012 to June 2013. aPWV and PWVβ were assessed in *n* = 780 (♀ = 49.4%) apparently healthy school children, aged 8–17 years. The project took part in a school setting. Written informed consent was obtained from children and/or parents. Children with chronic conditions like congenital heart defects were not involved in the study. The study protocol was approved by the ethical board of the Technical University of Munich (project number 5490/12). Study rationale, design, and further hemodynamic results are published elsewhere (14).

### Anthropometry and Blood Pressure Measurement

Body weight was measured wearing light clothes, without shoes, to the nearest 0.1 kg by trained staff (seca803, seca, Hamburg, Germany). Body height was measured to the nearest 0.1 cm with a stadiometer (seca799; seca, Hamburg, Germany). Standard deviation scores (SDS) for BMI were calculated and corresponding weight categories were defined according to German reference values (15).

BP was measured oscillometrically (Mobil-O-Graph® I.E.M., Stolberg, Germany) on the left arm, after participants rested for 10 min in supine position. According to German reference values (16), BP was categorized as normotensive (<90th percentile) or high normotensive (90th−95th percentile). Participants with BP above the 95th percentile were categorized as hypertensive (systolic and diastolic BP elevated) or with isolated systolic hypertensive values (ISH, only systolic BP elevated above the 95th percentile). As only one BP measurement was performed, this categorization is not identical to a clinical diagnosis of persistent hypertension.

### Discrimination

The total study population was divided in subgroups according to their weight status [*z*-score BMI SDS ≤1.282 indicating normal weight or underweight (N) vs. BMI SDS >1.282 representing overweight or obesity (O)], according to German reference values (15). Groups were compared regarding significant group differences in age, height, and peripheral blood pressure.

## PWV Measurement

### Sonographic Method—eTRACKING

**Figure 1:**
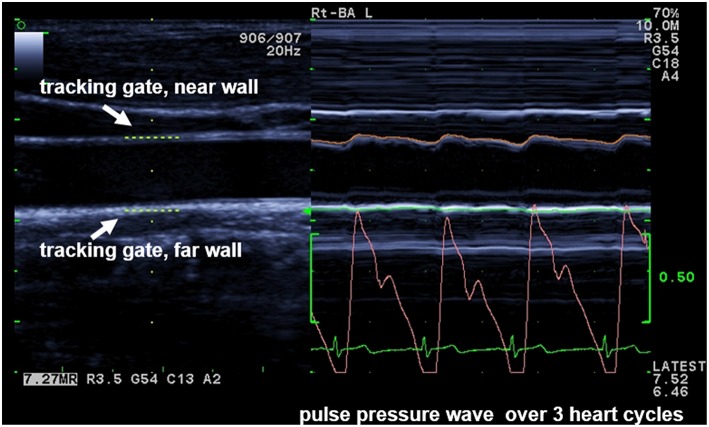
Ultrasound echo tracking (ET) PWV measurement at the common carotid artery.

With the eTRACKING system (ProSound Alpha 6® Aloka/Hitachi Medical Systems GmbH, Wiesbaden, Germany), the movement of the arterial wall during the cardiac cycle from diastole to systole is tracked with two digital tracking gates, placed at the CCA near and far wall to automatically follow wall motion and calculate diameter changes during heart cycles (17) (Figure 1). The method allows to calculate PWVβ from the time delay between two adjacent distension waveforms (18). An extensive and comprehensive description of underlying methodologies and assumptions to assess local arterial stiffness at the carotid artery is provided by Vriz et al. (18). Children were examined after a 15-min rest in supine position: the neck was slightly extended and the head was turned 45° opposite the site being scanned. PWVβ was measured 1 cm proximal to the bulb with a high-frequency linear array probe (5–13 MHz). Parameters were calculated as average values of four measurements (two measurements at each side). Interobserver variability of measurements is 1.37% (14).

### Oscillometric Method—ARCSolver

aPWV at the A. brachialis was assessed with the Mobil-O-Graph (I.E.M, Stolberg, Germany; ARCSolver method). The measurement is similar to an automated BP measurement. Depending on the upper arm circumference, an appropriate BP cuff (five different sizes) was chosen for each participant and attached on the left arm. The ARCSolver method consists of a three-level algorithm. First, single pressure waves are verified for plausibility. Second, a comparison of each single pressure wave is performed to detect artefacts. Third, a filtered, averaged pulse wave is derived and used to calculate the central aortic pulse wave (19) and hemodynamic parameters like central blood pressure or augmentation index.

### Statistics

All parameters (continuous variables) are expressed as mean ± standard deviation (SD) or as frequencies and percentages (non-continuous variables). The two methods were graphically compared by a Bland–Altman plot (20, 21). The limits of agreement (LoA) were defined as mean bias ± 2 SD, followed by the concordance correlation coefficient by Lin (ρc) (22–24) to evaluate reproducibility. Lin's ρc measures how well a measurement reproduces a gold standard measurement by quantifying the agreement between these two measures. It avoids inadequacies associated with different other tests (paired *t*-test, Pearson correlation coefficient *r*, coefficient of variation, interclass correlation coefficient) used in the context of assessing concordance between two alternative methods (25). It might also be superior to the Bland–Altman analysis and their LoA method (26) but is less used. Lin's ρc ranges from 0, indicating no substantial agreement, up to 1, representing perfect concordance between two methods. It should be interpreted similar to Pearson's correlation coefficient (27).

The independent *t*-test was performed to identify differences in aPWV and PWVβ by an increased BMI (>90th percentile). Data were analyzed using IBM SPSS statistical software for Windows, version 23 (SPSS, Inc, Chicago, IL, USA). Level of significance was defined as *p* < 0.05; all tests were two-sided.

## Results

### Anthropometry

Overall 780 children, 8–17 years, were included in the analysis (385 girls). Mean age was 11.61 ± 2.11 years. Mean BMI was 18.6 ± 3.4; *z*-score of BMI was 0.04 ± 1.1, indicating a study population with BMI values very close to the German reference population (15); 86.8% of participants were normal weight, and 14.2% were overweight, including 5.8% obese participants.

### Reproducibility

Interobserver variability for PWVβ was 1.37%, calculated by coefficient of variation (CV). The oscillometric measurement of aPWV, which is similar to a standard blood pressure measurement, is an operator-independent method (19, 28, 29). Therefore, no CV was calculated.

### Blood Pressure

Mean SBP was 116.29 ± 9.71 mmHg, and mean DBP was 68.09 ± 8.16 mmHg; 64.2% of all participants had normotensive or high normotensive blood pressure values; 16.8% had a single hypertensive blood pressure measurement, and 19% had an ISH measurement. The descriptive data are given in Table 1.

**Table 1:** Descriptive statistics of the study population (*n* = 780).

	**Mean**	**± SD**	**Min**	**Max**
Age [years]	11.61	2.11	8.00	17.92
Height [cm]	150.15	12.98	112.50	190.00
Weight [kg]	42.88	13.25	17.10	117.40
BMI [kg/m^2^]	18.61	3.38	11.93	33.33
BMI SDS	0.04	1.05	−3.65	2.95
SBP [mmHg]	116.29	9.71	85.00	152.00
SBP SDS	1.05	1.10	−3.20	5.15
DBP [mmHg]	68.09	8.16	44.00	93.00
DBP SDS	0.48	1.22	−3.10	4.29
cSBP [mmHg]	101.62	9.45	75.00	139.00
cSBP SDS	0.88	1.44	−3.02	7.29

*SD, standard deviation; BMI, body mass index; SDS, standard deviation score; SBP, systolic blood pressure; DBP, diastolic blood pressure; cSBP, central systolic blood pressure*.

### PWV

**Figure 2:**
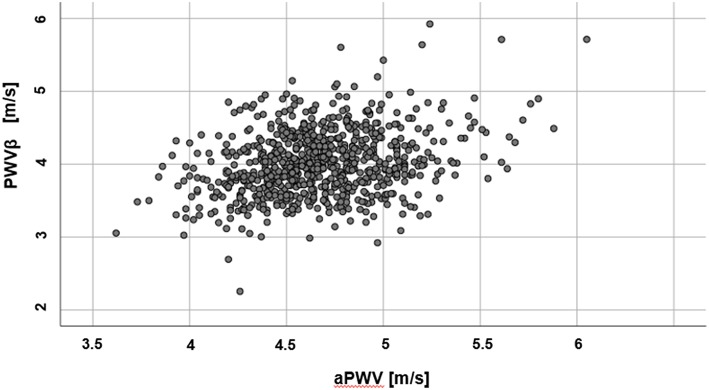
Scatter plot of measured PWVβ and aPWV values.

Mean values for PWVβ (ultrasound) were lower (4.01 ± 0.44 m/s) than aPWV (oscillometric, 4.67 ± 0.34 m/s). Results of the two methods differ by 0.66 ± 0.47 m/s (95% confidence interval of the difference: 0.62 to 0.69 m/s), *p* < 0.001. A scatter plot of PWVβ and aPWV is given in Figure 2.

**Figure 3:**
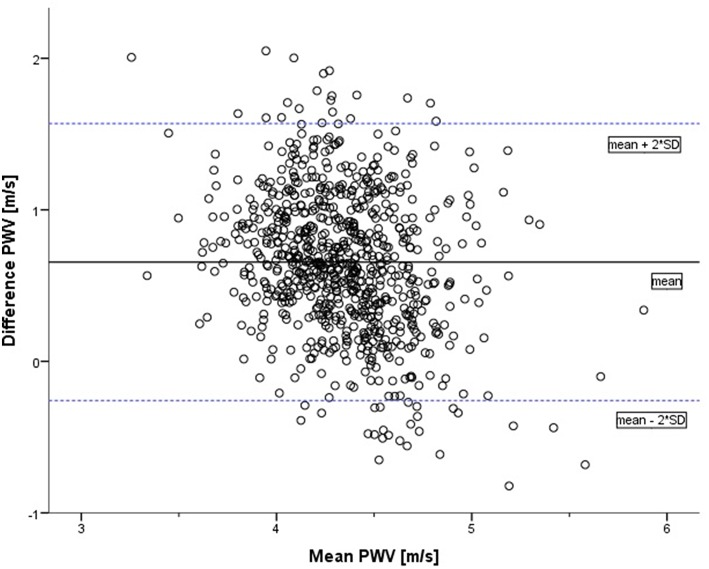
Bland–Altman plot of difference in pulse wave velocity (oscillometrically measured aPWV minus measurement by ultrasound PWVβ) against the mean of the two measurements (in m/s). The solid line represents the mean of the two methods: 0.66. The dotted lines represent the limits of agreement [mean + 2*SD = 1.57; mean – 2*SD = (−0.26)].

The Bland–Altman analysis (Figure 3) indicates the 95% LoA (−0.26 to 1.57). There was no evidence of systemic difference or obvious trend for the reproducibility of measurements to vary with their underlying mean value. The two methods do not consistently provide similar measures with a level of disagreement that includes clinically important discrepancies of up to 0.69 m/s. Lin's concordance correlation coefficient (ρc = 0.122; 95% CI: 0.093–0.150) represents a slight concordance between PWVβ and aPWV.

As obesity and overweight might influence PWV, Lin's concordance correlation coefficient was re-evaluated with the subgroup of non-overweight participants (*n* = 677). In this subgroup, concordance between the two measurements is even lower (ρc = 0.100; 95% CI: 0.070–0.130).

### Discrimination

There was no substantial difference between normal weight (N) and overweight (O) participants regarding age (*p* = 0.781) or peripheral blood pressure (SBP, *p* = 0.422/DBP, *p* = 0.318). Height [(N) 149.63 ± 13.00 cm vs. (O) 153.59 ± 12.39 cm, *p* = 0.004] and central systolic blood pressure [cSBP: (N) 100.95 ± 8.89 mmHg vs. (O) 105.98 ± 11.64 mmHg, *p* < 0.001] were higher in overweight children. SDS of peripheral blood pressure values indicate possible influences of height or age on this parameter.

**Figure 4:**
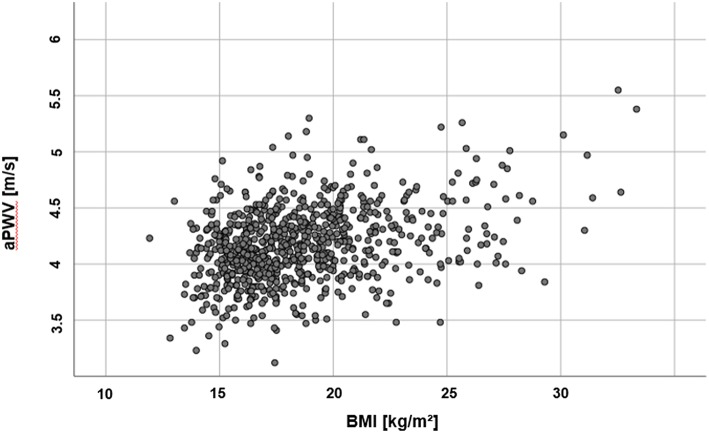
Scatter plot of measured aPWV (m/s) values and participants' BMI (kg/m^2^).

There was no difference in PWVβ between normal weight and overweight participants, *p* = 0.378. But aPWV was higher in overweight participants [(O) 4.81 ± 0.42 m/s] compared to normal weight participants [(N) 4.65 ± 0.32 m/s], *p* < 0.001 (Figure 4). This difference, −0.16, 95% CI [−0.25; −0.08] was significant *t*_(121)_ = −3.76, *p* < 0.001, with a medium-sized effect (30, 31), Cohen's *d* = 0.5. For further details, see Table 2. A discrimination between normal and altered vascular function was possible to detect with the oscillometric device (aPWV) but not with the ultrasound-based PWVβ.

**Table 2:** Differences between normal weight and overweight participants.

	**Normal weight ≤90th percentile (*****N*** **= 677)**		**Overweight** *****>*******90th percentile (*****N*** **= 103)**		***p*–value**	**Significance**
	**Mean**	**± SD**	**Mean**	**± SD**		
Age [years]	11.62	2.11	11.55	2.13	0.781	
Height [cm]	149.63	13.00	153.59	12.39	0.004	^*^
Weight [kg]	40.3	10.9	59.7	15.1	<0.001	^*^
SBP [mmHg]	116.03	9.30	118.07	11.98	0.100	
SBP SDS	1.03	1.08	1.13	1.24	0.422	
DBP [mmHg]	68.16	8.17	67.62	8.16	0.535	
DBP SDS	0.49	1.22	0.37	1.19	0.318	
cSBP[mmHg]	100.95	8.89	105.98	11.64	<0.001	^*^
cSBP SDS	0.77	1.36	1.61	1.77	<0.001	^*^
PWVβ [m/s]	4.01	0.44	4.05	0.46	0.378	
aPWV [m/s]	4.65	0.32	4.81	0.42	<0.001	^*^

*SD, standard deviation; SDS, standard deviation score; SBP, systolic blood pressure; DBP, diastolic blood pressure; cSBP, central systolic blood pressure; PWVβ, local pulse wave velocity, obtained by ultrasound; aPWV, pulse wave velocity of the Aorta, obtained oscillometrically*.

* *indicating significant results*.

## Discussion

Vascular alterations, which may lead to manifest CVD in future life, have a tremendous time delay between onset and obvious clinical appearance (32). Therefore, one goal of preventive measurements is to distinguish clearly between those persons with elevated risk and those without.

In this study, we measured PWV non-invasively with two alternative methods. For the first time, ultrasound-measured PWVβ was compared with oscillometrically measured aPWV in apparently healthy children and adolescents. The present study shows that aPWV is significantly higher than PWVβ with a clinically relevant level of disagreement, comparable to the study of Vriz et al. (18). Vriz et al. demonstrated a significant correlation of both techniques, rather than an agreement (33). In an adult population (mean age: 51.5 ± 14.1years), they reported a systematically higher aPWV than PWVβ (Δ1.4 m/s) (18). Both techniques are more and more applied to detect vascular alterations—though results might depend on the underlying setting (pediatric practice, pediatric cardiologist, specialized outpatient clinic). Therefore, the question of interchangeability of different methods comes up. A possible solution is to use individual cutoff values for each technique (18), but the more urgent question is whether a discrimination between normal PWV values and an altered vascular status is feasible with each method.

Overweight and obesity are known chronic conditions that influence hemodynamic parameters like PWV. The impact of obesity in adults on arterial stiffness seems to be evident: wall stiffening is accelerated in obese persons (34, 35). In childhood, current findings are controversial. Some studies demonstrated an increased PWV in obese children (17, 36–39) while others did not show any differences (34, 35) or reported a decreased PWV in obese children (40). Differences could be explained by substantial deviations in the studied populations (age, gender, comorbidities such as hypertension), but could also be due to the heterogeneity of different methods to assess vascular stiffness (36).

It does not seem to be realistic that obesity-caused vascular stiffening does not start before adulthood. In autopsy studies, fatty streaks and pro-atherosclerotic lesions were present in children between 1 and 15 years (41–43). A recent meta-analysis by Hudson et al. (44) revealed increased arterial stiffening in obese children, especially in central arteries. The authors emphasized a variation in arterial stiffness between different vascular regions.

In our study a distinction between normal weight and overweight children in terms of altered vascular function and stiffening was possible by aPWV but failed with PWVβ. One explanation is the different location of measurements (aPWV: Aorta; PWVβ: A. carotis). Along the arterial tree, elastic properties of the vessel vary within each region. Proximal arteries are more elastic whereas stiffness increases in distal arteries. Majesky stated that “[…] different vessels, or even different segments of the same vessel, are composed of smooth muscle cell populations that arise from distinct sources of progenitors, each with its own unique lineage and developmental history” (45). This vascular smooth muscle diversity might be the key element to different blood vessel functions and the susceptibility to risk factors. Even though the influence of risk factors should have comparable effects in all arterial beds, it could be demonstrated by DeBakey and Glaeser (46) that each vascular field has its own distinctive response to the atherogenic process. Moreover, little is known about the impact of maturation in adolescence, as the lineage-specific boundaries of smooth muscle cells “[…] may shift their relative positions during vascular growth and aging.” Vascular changes resulting in higher PWV might occur later in the carotid artery than in the Aorta. Vriz et al. (18) emphasize that aortic stiffness and carotid stiffness do not seem to be completely interchangeable predictors in high-risk patients, although both stiffness parameters provide similar information on the effect of aging on elastic arteries.

Another explanation could be that the technique to obtain PWVβ is less sensitive for subtle changes caused by earliest vascular alterations. The question remains, if vascular alterations are stable and predictive for future adverse cardiovascular phenotypes, which could already be demonstrated for other vascular parameters like flow-mediated dilatation (FMD) (47). Therefore, long-term follow-up studies (minimal duration: 10 years) are mandatory to establish PWV in a general pediatric setting as a useful early diagnostic tool.

In conclusion, PWVβ measured by ultrasound and aPWV obtained oscillometrically do not provide similar information and cannot be compared interchangeably. The level of disagreement is not only statistically significant but also clinically relevant: The range of PWVβ is from 2.91 to 5.92 m/s in girls (age 8–17) and 2.25 to 5.71 m/s in boys (age 8–17), and for aPWV, it is 3.62 to 5.45 m/s in girls (age 8–17), and 3.79 to 6.08 m/s in boys (age 8–17). The mean differences between PWVβ and aPWV is 0.61 m/s, a difference in speed that is higher than one SD in this population (mean PWVβ ± SD: 4.01 ± 0.44 m/s and mean aPWV: 4.67 ± 0.34 m/s). In adults, an increase in aPWV of 1 SD is associated with a 1.35 higher risk for coronary heart disease, 1.54 higher risk for stroke, and 1.45 higher risk for CVD (48). Therefore, measuring PWV already in children is a useful tool to detect vascular alterations at an early point.

In clinical routine, the sonographic PWV measurement is an interesting alternative to other methods. In addition to IMT and other vascular parameters, carotid PWVβ could be measured in only one examination, with one device. However, the ultrasound technique for PWVβ is possibly less sensitive in detecting earliest vascular changes.

## Ethics Statement

Ethics Commission, Medical Faculty, Technical University of Munich Projet number 5490/12.

## Author Contributions

JE: study concept, data analysis, and writing the manuscript. HW: study concept, data aquisition, and editing the manuscript. MM: data aquisition and reviewing the manuscript. KP: data analysis and reviewing the manuscript. RO: study concept and reviewing the manuscript.

### Conflict of Interest Statement

The authors declare that the research was conducted in the absence of any commercial or financial relationships that could be construed as a potential conflict of interest.
